# Radiomics in Cardiac Computed Tomography

**DOI:** 10.3390/diagnostics13020307

**Published:** 2023-01-13

**Authors:** Isabelle Ayx, Matthias F. Froelich, Stefan Baumann, Theano Papavassiliu, Stefan O. Schoenberg

**Affiliations:** 1 Department of Radiology and Nuclear Medicine, University Medical Center Mannheim, Heidelberg University, Theodor-Kutzer-Ufer 1-3, 68167 Mannheim, Germany; 2 First Department of Medicine-Cardiology, University Medical Center Mannheim, Theodor-Kutzer-Ufer 1-3, 68167 Mannheim, Germany; 3 DZHK (German Centre for Cardiovascular Research), Partner Site Heidelberg/Mannheim, Mannheim, Germany

**Keywords:** cardiac computed tomography, cardiac computed tomography angiography, radiomics, texture analysis, cardiovascular disease

## Abstract

In recent years, there has been an increasing recognition of coronary computed tomographic angiography (CCTA) and gated non-contrast cardiac CT in the workup of coronary artery disease in patients with low and intermediate pretest probability, through the readjustment guidelines by medical societies. However, in routine clinical practice, these CT data sets are usually evaluated dominantly regarding relevant coronary artery stenosis and calcification. The implementation of radiomics analysis, which provides visually elusive quantitative information from digital images, has the potential to open a new era for cardiac CT that goes far beyond mere stenosis or calcification grade estimation. This review offers an overview of the results obtained from radiomics analyses in cardiac CT, including the evaluation of coronary plaques, pericoronary adipose tissue, and the myocardium itself. It also highlights the advantages and disadvantages of use in routine clinical practice.

## 1. Introduction

Due to the improvement in computed tomography technology, the importance of additional diagnostic tools in the field of cardiovascular imaging has been recognized by medical societies, leading to a readjustment of guidelines and recommendations for cardiac CT angiography (CCTA) [1]. In the sense of a so-called rule-out strategy, patients with low to intermediate pretest probability should receive an initially noninvasive workup [1]. CCTA provides high diagnostic accuracy for detecting significant coronary artery stenosis of more than 50% but also offers an optimal exclusion of obstructive coronary artery disease with a negative predictive value of 99% [2,3]. In recent years, plaque characterization made the additional identification of high-risk plaques in dedicated CCTA possible [4]. Recently published, the DISCHARGE trial revealed a lower procedure-related complication rate in patients receiving prior CCTA to invasive coronary angiography [5].

Nevertheless, the main focus in daily routine lies in the determination of coronary artery stenosis. However, the introduction of radiomics to the medical society, as an opportunity to analyze quantitative data by extracting numerous features from images—invisible to the human eye, offers new potential for cardiac CT imaging [6]. These numerous features of the region of interest (ROI) provide additional information and new possibilities in the rising big data trend in healthcare. The first promising results could be seen in oncologic imaging, allowing for example the prediction of disease-free survival in non-small cell lung cancer [7] or the prediction of patients’ survival with metastatic colorectal cancer by a CT-based analysis of whole liver tumor burden [8]. Apart from oncologic research, radiomics analysis shows likewise promising results for example in providing a tool for the quantification of idiopathic pulmonary fibrosis in high-resolution CT [9] and as a potential imaging biomarker in periaortic adipose tissue in arteriosclerosis [10].

In the last years, radiomics analysis showed incremental value in the cardiovascular imaging field [11,12]. This review article summarizes the application of radiomics analysis in the cardiac field and outlines challenges and future opportunities.

## 2. Basic Principles of Radiomics

**Figure 1:**
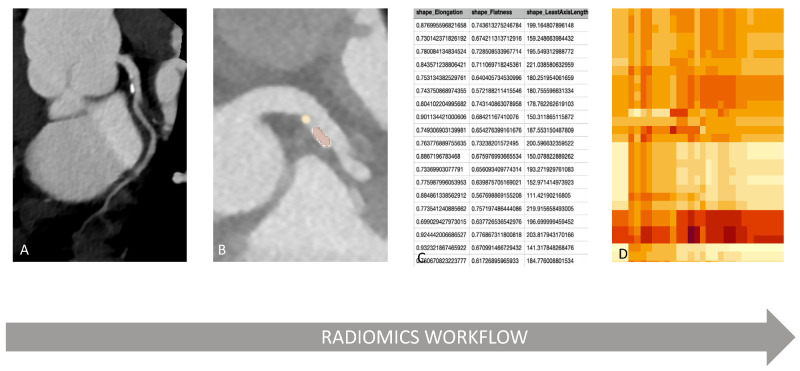
**Example of radiomics workflow**. A high-quality CCTA image (**A**) is the foundation for radiomics application. After segmentation of the region of interest (plaques marked in yellow and orange (**B**), features can be extracted using dedicated software (parameters of various radiomics features (**C**). Features are selected in dependence on i.e., clinical parameters and models can be created further, for example by creating a heatmap (**D**).

Radiomics analysis uses methods from the field of artificial intelligence to quantify textural information by extracting the spatial distribution of Hounsfield or signal intensities and relationships. In contrast to previously known computer-aided diagnosis and detection systems (CAD), radiomics analysis is not a standalone system to deliver a single answer but a process for extracting numerous quantitative data from digital images and mining the data for hypothesis generating [6]. It starts by defining a region or a volume of interest (ROI/VOI) in image data [6]. However, the underlying image data may influence radiomics analysis. Hence, image acquisition should be standardized and recorded. High image quality minimizes feature variability as one of the main quality requirements for successful radiomics analysis is the combination of optimal spatial resolution and signal-to-noise ratio [13,14,15]. The segmentation of ROI/VOI can be carried out manually, semi-automatically, or fully automatically. Through dedicated software [16], features can be extracted from the ROI/VOI and can be analyzed further statistically (Figure 1).

Radiomics features can be divided into different subgroups. First-order features describe the distribution of voxel intensities independently of their spatial relationship. Texture features on the other hand take the spatial relationship into account and characterize the heterogeneity of the underlying lesion. Mainly five different types of texture features are used in the routine, defined by gray-level cooccurrence matrix (GLCM), gray-level run-length matrix (GLRLM), gray-level size zone matrix (GLSZM), gray-level dependence matrix (GLDM), and neighboring gray-tone difference matrix (NGTDM). Additionally, shaped-based features further quantify the spatial complexity, and finally, transform-based features convert spatial information into specific new information or filter specific information [17,18,19,20]. These features can be correlated with clinical parameters and may offer a more precise analysis of the image data, possibly influencing therapies and estimating outcome prediction.

## 3. Radiomics Analysis in Cardiac Imaging

### 3.1. Radiomics Analysis of Coronary Artery Plaques

Beyond purely estimating the degree of coronary artery stenosis, CCTA can detect different plaque characteristics. Four different plaque characteristics have been linked to the development of major adverse cardiovascular events, namely low-density plaques, positive remodeling, spot calcification, and napkin ring sign (NRS) [4]. One study revealed low-attenuation plaque burden as the strongest predictor of fatal or nonfatal myocardial infarction [21]. Even in nonobstructive lesions, these distinct parameters correlated positively with adverse outcomes [22,23,24]. However, in clinical settings, these parameters are not always easy to determine due to time-consuming calculations. Therefore, Kolossvary et al. investigated in 2017 the possibility of radiomics analysis to distinguish plaque with and without NRS. They included 30 patients with NRS plaques, who were referred to CCTA due to stable chest pain. Additionally, they matched 30 plaques without napkin ring signs with similar luminal obstruction, localization, degree of calcification, and imaging parameters. They defined eight different conventional quantitative metrics (namely lesion length and volume, mean plaque burden, lumen area stenosis, vessel wall remodeling index as well as mean, minimal, and maximal plaque attenuation), but none of these parameters showed a significant difference between NRS and non-NRS plaques. On the contrary, 4440 radiomics parameters were calculated for each manually segmented atherosclerotic lesion. Out of these parameters, 20.6% (916 parameters) showed a significant difference between both plaque types (all *p* < 0.0012). The five most differentiating parameters were short-run low-gray-level emphasis, long-run low-gray-level emphasis, surface ratio of component 2 to total surface, long-run emphasis, and surface ratio of component 7 to total surface (AUC values: 0.918; 0.894; 0.890; 0.888 and 0.888, respectively). Hence, radiomics analysis of coronary artery plaque outperformed conventional parameters in identifying NRS plaques in cardiac CT [25].

Following this study, Kolossvary et al. went one step further in 2019 by investigating the possibility of radiomics analysis to outperform conventional assessment of CCTA to identify invasive and radionuclide imaging markers of plaque vulnerability. The 25 patients, who underwent CCTA, sodium-fluoride positron emission tomography (PET), intravascular ultrasound (IVUS) and optical coherence tomography (OCT), were prospectively included in their study, leading to the identification of 44 plaques. For each invasive and radionuclide imaging marker the best conventional and the best radiomics parameter for identification was identified: IVUS-attenuated plaques could be identified by non-calcified plaque volume on CCTA as well as with fractional box-counting dimension of high attenuation voxels in radiomics analysis (AUC 0.59 and 0.72, respectively, *p*-value < 0.001). OCT identified thin-cap fibroatheroma correlated with the presence of low attenuation in CCTA and the fractal box-counting dimension of high attenuation voxels in radiomics analysis (AUC 0.66 and 0.80, respectively, *p*-value < 0.001), [^18^F]NAF-PET positivity with the presence of two out of four high-risk features in CCTA and surface of high attenuation voxels in radiomics analysis (0.65 and 0.87, respectively, *p*-value < 0.001). Summarizing these results, radiomics analysis outperformed conventional parameters for the identification of plaque vulnerability significantly [11].

Both studies demonstrate the possibility of radiomics analysis to increase the diagnostic accuracy of CCTA in the identification of vulnerable plaque characteristics.

In 2021, another study investigated the performance of the radiomics analysis of CCTA in identifying hemodynamically significant coronary artery stenosis. Li et al. compared conventional parameters as well as radiomics parameters, both derived from CCTA, in 149 patients (174 plaques with a stenosis degree between 30% and 90%) with the gold standard of invasive fractional flow reserve (FFR), building a randomly selected training and validation model. Non-calcified plaque (NCP) volume, lesion length, spotty calcification, remodeling index (RI), NRS, and stenosis degree were recorded as conventional parameters. In all, 58 out of 523 radiomics features correlated with hemodynamically significant stenosis (*p* < 0.05), whereas 56 yielded an AUC above 0.6. Out of these parameters, 14 parameters were used to build a radiomics model: NCP volume, NRS, RI, and spotty calcification for the conventional model. In the training and validation set, AUC showed an improvement (0.71 and 0.82 for training, 0.70 and 0.77 for validation; conventional and radiomics model, respectively) but not a statistical significance (*p* = 0.58) [26].

### 3.2. Radiomics Analysis of Pericoronary Adipose Tissue

Vascular inflammation has been linked to the structural changes and remodeling of perivascular adipose tissue (PVAT) [27]. The recently described Perivascular Fat Attenuation Index (FAI) outlines the inflammation-induced increase in CT attenuation values and counts as a strong predictor of major adverse cardiovascular events (MACE) [28,29]. The texture analysis of perivascular adipose tissue is meant to look beyond the FAI and possibly define imaging biomarkers. In line with this hope, Oikonomou et al. investigated 2019 the radiomics profile of PVAT remodeling for the improvement of cardiac risk prediction. Their first study obtained adipose tissue biopsies from 167 patients and correlated radiomics features to gene expression representing inflammation, fibrosis and vascularity. Tissue inflammation, outlined by tumor necrosis factor-alpha (TNFA) expression, was correlated best with adipose tissue wavelet-transformed mean attenuation. However, fibrosis and vascularity could be comparable or better reflected by higher-order texture features than mean attenuation. Additionally, they analyzed radiomics features in 101 patients who experienced MACE within 5 years after CCTA and 101 matched controls. The fat radiomics profile (FRP) was able to improve MACE prediction significantly compared to traditional risk factors such as coronary calcium score, coronary stenosis, and high-risk plaque features (*p* < 0.001). In line with these results, FRP was significantly higher in patients with acute myocardial infarction in comparison to matched controls (*p* < 0.001), outlining adverse PVAT remodeling [12]. Concordant with these findings, Lin et al. demonstrated that texture- and geometry-based radiomics parameters were able to distinguish patients with myocardial infarction and with stable or no coronary artery disease. Radiomics analysis hence provides information that was not captured by the attenuation-based model [30].

Another study compared the power of prediction of the future acute coronary syndrome within 3 years after CCTA of radiomics analysis of pericoronary adipose tissue to conventional plaque characteristics. Shang et al. identified 90 patients with acute coronary syndrome (ACS) within 3 years after CCTA and segmented pericoronary adipose tissue surrounding the culprit lesion, as well as notated 14 different conventional plaque characteristics. In 90 matched controls, the most severe stenotic lesion was evaluated in the same manner. Out of both parameter groups, a radiomics score (14 features) and a plaque score (minimal lumen diameter and high-risk plaque) were built. The radiomics score outperformed the plaque score significantly in identifying patients with future ACS within 3 years (AUC = 0.826, 0.811 radiomics score; AUC = 0.699, 0.640 plaque score, in training and test set, respectively) [31].

### 3.3. Radiomics Analysis of Left-Ventricular Myocardium

Magnetic resonance imaging (MRI) is commonly used for myocardial analysis [32,33,34]. However, research has also focused on the detection of myocardial scar and perfusion defects in cardiac CT [35]. In comparison to MRI, cardiac CT analysis offers the potential advantage of combined analysis of coronary artery stenosis and correlating myocardial scar. Nevertheless, CT suffers from insufficient contrast to the noise ratio of delayed iodine-enhanced scans [36]. Additionally, the delayed iodine-enhanced scan is an additional scan to CCTA leading to higher radiation exposure. Hence, several studies investigated in the recent past the potential of radiomics analysis to overcome these limitations. Antunes et al. already demonstrated in 2016 on a small patient collective of seven patients the potential of radiomics parameters to differ between patients with normal and scarred myocardial tissue post myocarditis. They investigated first-order radiomics parameters of left ventricular myocardium on normal and scarred myocardial tissue on the basal scan, angiographic scan and delayed iodine-enhanced scan, as well as on an ECV map calculated from myocardial and blood pool Hounsfield units (HU). The first-order parameter energy was the best parameter for differentiation between normal and scarred tissue in all scans (*p* < 0.001). Entropy, kurtosis, mean, and root mean square error were also able to distinguish between both tissues significantly on angiographic scans [37].

Following these results, Hinzpeter et al. illustrated in 2017 the feasibility of radiomics texture analysis for the differentiation of healthy from acutely infarcted myocardium in cardiac CT on 20 patients diagnosed with acute myocardial infarction (MI) and 20 matched controls. They were able to define different radiomics features for distinguishing both groups: Kurtosis was the most accurate first-level feature (AUC: 0.78, *p* = 0.002); correlation the most accurate second-level feature (AUC: 0.81, *p* = 0.002); and SRHGE the most accurate third-level feature (AUC: 0.82, *p* = 0.001) on 5 mm slice thickness short axis reconstruction CT images. In addition, they investigated the influence of different slice thicknesses of CT reconstruction on texture analysis, demonstrating a 5 mm slice thickness as the most accurate [38].

Going one step further, Mannil et al. revealed the potential of texture analysis in detecting myocardial fibrosis on non-contrast low radiation dose cardiac CT images being visually invisible. They included 27 patients with acute myocardial infarction, 30 patients with chronic myocardial infarction, and 20 patients with no cardiac abnormalities in their study. A visible differentiation between the groups was not possible. Texture analysis revealed moderate accuracy for differentiation between the three groups. However, improved accuracy was achieved when comparing patients with acute or chronic myocardial infarction and no cardiac abnormalities, indicating a possible overlap between texture features in infarcts of different ages (AUC 0.78, sensitivity 86%, specificity 81%) [33].

In all, 154 patients receiving CCTA and SPECT myocardial perfusion imaging were included in a study by Shu et al. for the development and validation of a CT-based radiomics machine learning model for the prediction of chronic myocardial ischemia. These patients were divided into a training set (n = 107) and into a test set (n = 47). Radiomics features were extracted from the left ventricular myocardium and feature reduction identified eight relevant features. For these eight features, multivariable logistic regression was used to create a radiomics signature. In addition, a radiomics nomogram was created based on a predictive model, derived from machine learning in combination with clinically related factors. This nomogram was then validated on the test set and an additional validation set, consisting of 49 patients (18 with chronic myocardial ischemia) from another medical center. Using a decision curve analysis, the clinical feasibility of the nomogram was demonstrated, and a significant difference could be detected between patients in a high or low-risk group. This study allowed the combination of radiomics with machine learning algorithms to differentiate between chronic myocardial ischemia and healthy myocardium in CCTA images and was even able to identify the higher risk population of chronic myocardial ischemia [39].

Going beyond the visualization of focal scarred myocardium, Esposito et al. aimed to correlate radiomics features generated from late iodine enhancement cardiac CT images of non-scarred remote myocardium from patients with recurrent ventricular tachycardia (rVT) with left ventricular (LV)- function (ejection fraction, end-diastolic diameter, and diastolic function of LV determined by transthoracic echocardiography), LV-remodeling, and underlying cardiac disease. In all, 48 patients with rVT were included in their study consisting of five patients with idiopathic ventricular tachycardia, 23 with post-ischemic dilated cardiomyopathy, nine with idiopathic dilated cardiomyopathy, and 11 with scars from previous myocarditis. LV systolic and diastolic function was assessed using echocardiography. A cardiac CT scan with non-contrast, angiographic and late iodine-enhanced scan was used to determine the end-diastolic volume (EDV) and extracellular volume (ECV). Scars were identified as areas of wall thinning or areas of late iodine enhancement and segmented for radiomics analysis. Different features (energy, HU mean, and HU median) correlated with ECV (*p* < 0.05). ECV and various radiomics features correlated with EDV. Additionally, two features (standard deviation (SD) and mean absolute deviation (MAD)) correlated with diastolic function. Both features presented significantly lower values in patients with idiopathic ventricular tachycardia and in patients with scars from healed myocarditis in comparison to dilated cardiomyopathy. Hence, they proved that myocardial heterogeneity was associated with systolic and diastolic function as well as LV dilatation and has the ability to distinguish different patterns of structural remodeling [40].

In line with these results, Kay et al. created an end-to-end pipeline, consisting of automated segmentation, radiomic feature extraction, and machine learning, to predict MRI-proven high-risk left ventricular hypertrophy (LVH) phenotypes on non-contrast cardiac CT. Out of a group of 1982 participants they identified 224 participants with high-risk LVH in cardiac MRI. All patients underwent additional non-contrast cardiac CT. Using an automated algorithm for segmentation of the left ventricle in cardiac CT, they extracted 107 radiomics features. Different feature selection models were used to access the probability of high-risk LVH as defined in cardiac MRI not only using the non-contrast cardiac CT scan but also gender, height, and body surface area. Additionally, they evaluated the pipeline in an internal validation set and concluded, that there is underutilized data embedded in non-contrast cardiac CT which could lead to the identification of high-risk individuals without the need for additional radiation exposure or imaging [41].

**Figure 2:**
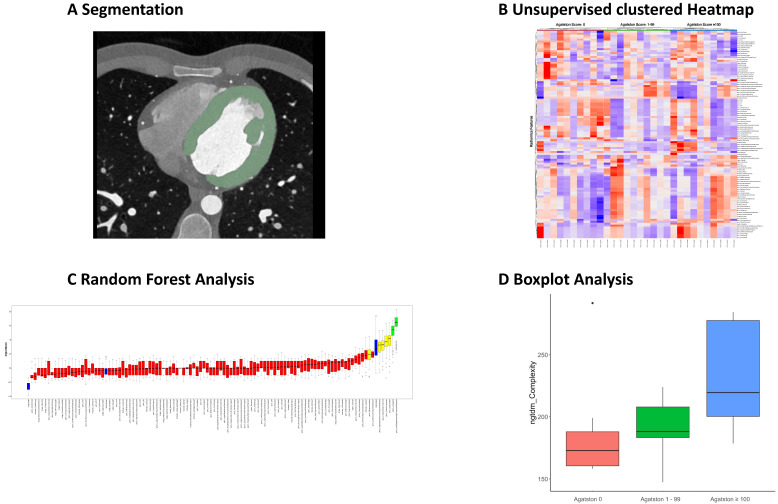
**Radiomics analysis of left ventricular myocardium**. Segmentation of left ventricular myocardium in dedicated software (**A**). Feature extraction and connection of clinical parameter (Agatston Score), visualized in a Heatmap (**B**). Random Forest feature selection to identify differentiating features between different Agatston Scores (**C**). Selected differentiating feature visualized in boxplot in dependence of Agatston Score (**D**). Data derived from Ayx et al. [41].

Recently, the unique possibility of CCTA to allow the estimation of coronary artery sclerosis and the potential detection of myocardial fibrosis was investigated in our institution. Radiomics parameters of left ventricular myocardium of patients with and without coronary artery calcifications measured by Agatston Score were compared in three different groups: as a training set, patients with an Agatston Score of 0 were compared to patients with an Agatston Score of ≥100. As a validation set, patients with an Agatston Score between 1–99 were chosen. Random forest selection allowed differentiation between the training groups by four different parameters (namely GLDM Small Dependence High Gray Level Emphasis, GLCM Cluster Shade, GLRLM Long Run Low Gray Level Emphasis, and NGTDM Complexity). For internal validation purposes, these four features were additionally investigated in the Agatston Score group of 1–99. Boxplots were used to visualize the distribution of the mean value of each feature in dependence on the Agatston Score. The Agatston Score group of 1–99 was settled between the training set group, indicating a change of texture parameters associated with the severity of coronary artery calcification. The differentiating feature complexity is a parameter for the heterogeneity of the underlying tissue. In this study, the value increased with increasing Agatston Score. In line with these findings, the feature cluster shade also increased depending on the Agatston Score and is known to be a measurement of skewness and uniformity. This increase indicates a greater asymmetry around the mean. Both features seem to indicate a more heterogenous structure of the left ventricular myocardium in patients with coronary artery calcification due to a potential remodeling effect. In total, these preliminary findings on a small patient population outline the potential severity-associated effect of coronary artery calcifications on the left ventricular myocardium as a possible correlate for a structural change (Figure 2) [42].

In addition, Cavallo et al. outlined a successful CCTA-based radiomics approach to identify left ventricular remodeling in patients with arterial hypertension. They included 83 patients with arterial hypertension and 75 control patients who underwent cardiac CT and segmented the left ventricular myocardium. In total, 377 radiomics features were extracted. The dataset was divided into two parts using a 7:3 ratio for training the classification model and afterward for testing and evaluating the model performance. Models with an accuracy higher of 60% were selected and finally, an Ensemble Machine Learning (EML) score was calculated. The EML score correlated to LV septum width (0.53, *p*-value < 0.0001). As the authors defined LV septum width as a surrogate of myocardial remodeling, they considered the EML score as a potential tool for evaluating myocardial remodeling [43].

### 3.4. Radiomics Analysis of Cardiac Mass

First attempts have also been made in mass characterization on cardiac CT images. In 2019, Nam et al. investigated 39 periprosthetic masses in 34 patients suspected of periprosthetic valve obstruction clinically. All patients underwent cardiac computed tomography and were clinically suspected of prosthetic valve obstruction (PVO). The final cause of PVO was then identified using redo-surgery and follow-up imaging as a standard reference. In 20 cases pannus could be finally diagnosed, in 11 cases thrombi, and in eight cases vegetations. Visual analysis of differentiating pannus from other abnormalities was compared to a radiomics score. Radiomics analysis was able to differentiate between pannus and other tissue (AUC = 0.876), leading to the combination of visual and radiomics assessment to outperform the visual assessment alone [44]. In line with these results, Qian et al. proved in 2021, that radiomics analysis is better than conventional assessment for differentiation of cardiac myxomas and cardiac thrombi on cardiac CT (AUC = 0.926 and 0.878, respectively). They included 109 patients who had cardiac myxoma (n = 59) and cardiac thrombi (n = 50) in their retrospective study. Two radiologists documented and compared the lesion characteristics in cardiac CT. Afterwards, all patients were allotted to a primary group or a validation group using a ratio of 7:3. Robust features were selected using univariate analyses and least absolute shrinkage and selection operator, leading to an identification of eight selected radiomic features consisting of five first-order features and three higher order features. This was compared to visual assessment using an independent clinical model with parameters such as calcification, location, enhanced CT value, and uniformity of density, as well as pathologies in the adjacent structures. This clinical model was outperformed in distinguishing cardiac myxoma and cardiac thrombi through a radiomics signature [45]. In addition, Chun et al. identified 95 patients with valvular heart disease and filling defects in the left atrial appendage on cardiac CT. The filling defects were classified as thrombus (n = 25) or as stasis (n = 70) using transesophageal echocardiography or cardiac surgery. Additional to radiomics analysis using a three-dimensional segmentation of filling defects on early-phase CT, the ratio of Hounsfield units within the filling defects was measured and compared to those in the ascending aorta in early and in late phases. In radiomics analysis, eight wavelet-transformed radiomics features were lower in thrombus than in stasis (*p* < 0.001). Comparing these findings with conventionally measured HU values, radiomics analysis (namely wavelet_LHL and wavelet_LLH) could differentiate better between thrombi and circulatory stasis in patients with left atrial appendage filling defects [46].

### 3.5. Radiomics Analysis of Periaortic Adipose Tissue

However, cardiac imaging goes beyond purely investigating the heart itself. As already mentioned above, perivascular adipose tissue, surrounding blood vessels immediately, is known to be metabolically active [27]. Various studies in the last years have focused on the effect of the volume and density of periaortic adipose tissue; Lehman et al. could identify a possible connection between periaortic adipose tissue, metabolic risk factors, and vascular calcification. They included all patients with an interpretable scan for periaortic adipose tissue who were free of established cardiovascular disease from the Framingham Heart Study Offspring cohort and quantified adipose tissue semi-automatically to calculate the adipose volume surrounding the thoracic aorta over a 6.75 cm column. The volume of the periaortic adipose tissue correlated with the body mass index (BMI), waist circumference (WC), hypertension, lower HDL, serum triglycerides, impaired fasting glucose, and diabetes even after adjustment for BMI and WC. Additionally, thoracic periaortic adipose tissue was associated with abdominal aortic calcification and coronary artery calcification [47]. In line with these results, Zhu et al. demonstrated in a volume-based approach an association of periaortic adipose tissue and visceral adipose tissue with coronary artery atherosclerosis in 2021 [48]. However, studies regarding the radiomics texture analysis of periaortic adipose tissue are still very limited. One preliminary study recently analyzed texture features of periaortic adipose abdominal tissue in dependence of local aortic calcifications. In total, 30 patients were selected, building two groups of with and without abdominal aortic calcification. Adipose tissue was segmented within a ring of 5 mm surrounding the abdominal aorta ranging from below the junction of the renal arteries to the aortoiliac bifurcation. Random forest feature selection identified seven radiomics features, that showed a significant difference between both groups: GLCM Contrast, GLCM Difference Variance, GLCM Difference Average, GLCM Difference Entropy, GLCM Joint Entropy, GLSZM Gray Level Variance, and GLCM Maximum Probability. These findings revealed a prediction of the presence of aortic calcifications using radiomics texture analysis of periaortic adipose tissue, possibly indicating a potential imaging biomarker for atherosclerosis [10]. Further studies in this field must follow to investigate periaortic adipose tissue in the thoracic aorta with a potential local and down streaming effect.

Summarizing literature, Table 1 offers an overview of important studies related to cardiovascular radiomics analysis.

## 4. Challenges and Limitations of Radiomics Analysis

Even though radiomics analysis shows promising results in cardiac CT imaging, the implementation of radiomics analysis in clinical routine has not been fulfilled yet. Radiomics analysis is susceptible to many technical factors and hence lacks reproducibility. Different studies describe the influence of different manufacturers as well as various image acquisition parameter settings on feature stability [49]. Not only the choice of contrast agent and media phases but also underlying parameters such as tube voltage, reconstruction kernel, and slice thickness influence the reproducibility of feature analysis [19,50]. Even post-processing tools such as segmentation methods [51] and feature extraction software [52] impede feature stability. As long as these factors significantly influence radiomics analysis, a sufficient approach to implement this tool in decision-making in clinical practice is limited. In cardiac imaging, various parameters can be influenced from the very beginning and may lead to a more stable radiomics analysis between different centers, if widely recognized and adapted in clinical routine. Additionally, segmentation methods and feature extraction software could be standardized to reduce these challenges. Recently, promising results are shown by the implementation of photon-counting detector CT (PCCT) leading to better signal-to-noise ratio, high spatial resolution, and lower beam-hardening artifacts [53]. A preliminary study revealed a comparability of first-order radiomics features between PCCT and conventional energy-integrating CT but differences in higher-order features of the left ventricular myocardium. These differences might be due to the impact of the new technology and may push cardiac radiomics analysis in the future [54]. However, whether PCCT will revolutionize radiomics analysis and finally lead to implementation in clinical practice must be proven first. In total, further work will be needed to find a way for stabile radiomics analysis and the potential of reproducibility between different centers, before radiomics analysis can be widely and reliably used in clinical routine.

## 5. Potential Outlook in the Future

Despite the challenges of radiomics analysis, the radiomics of cardiac CT will find its way into the clinical routine in one way or another. The strength of feature analysis in cardiac CT is to go beyond the pure visual assessment of image data and to add quantitative information. This quantitative information will be needed for future clinical decision-making as well as for exploring biomarkers in prevention and personalized cardiovascular medicine. Even though this is an ambitious goal, the foundation is already laid, and future work must and will overcome the limitations to implement radiomics of cardiac CT in clinical routine.

## Figures and Tables

**Table 1:** Overview of radiomics analysis in cardiac imaging.

**Radiomics analysis of coronary artery plaques**	Kolossvary et al. *Circ: Cardiovascular Imaging* 2017 [25]	Ability of radiomics features to differentiate between plaques with and without napkin ring signs
	Kolossvary et al. *European Heart Journal–Cardiovascular Imaging* 2019 [11]	Radiomics analysis outperformed conventional assessment in terms of plaque vulnerability
	Li et al. *European Journal of Radiology* 2021 [26]	Radiomics analysis is better at identifying hemodynamically significant coronary artery stenosis than conventional parameters
**Radiomics analysis of pericoronary adipose tissue**	Oikonomou et al. *European Heart Journal* 2019 [12]	Radiomics profile of PVAT remodeling for improvement of cardiac risk prediction
	Lin et al. *JACC: Cardiovascular Imaging* 2020 [30]	Differentiation of patients with myocardial infarction and with stable or no coronary artery disease by radiomics features
	Shang et al. *Eur Radiol* 2022 [31]	Power of prediction of future acute coronary syndrome within 3 years by radiomics analysis
**Radiomics analysis of left-ventricular myocardium**	Antunes et al. *Annu Int Conf IEEE Eng Med Biol Soc.* 2016 [37]	Radiomics features could differ between patients with normal and scarred myocardial tissue post myocarditis
	Hinzpeter et al. *PLoS ONE* 2017 [38]	Feasability for differentiation of healthy from acutely infarcted myocardium by radiomics analysis
	Mannil et al. *Investigative Radiology* 2018 [33]	Radiomics features detecting myocardial fibrosis on non-contrast low radiation dose CT
	Shu et al. *J. Nucl. Cardiol.* 2022 [39]	Radiomics machine learning model for prediction of chronic myocardial ischemia
	Esposito et al. *Mol Imaging Biol* 2018 [40]	Radiomics analysis could distinguish different patterns of structural remodeling in patients with rVT
	Kay et al. *Circ: Cardiovascular Imaging* 2020 [41]	Prediction of MRI-proven high-risk left ventricular hypertrophy phenotypes on non-contrast cardiac CT through radiomic analysis
	Ayx et al. *Diagnostics* 2022 [42]	Potential detection of myocardial fibrosis by radiomics features in dependence on coronary artery sclerosis
	Cavallo et al. *Diagnostics* 2022 [43]	CCTA-based radiomics approach to identify left ventricular remodeling in patients with arterial hypertension
**Radiomics analysis of cardiac mass**	Nam et al. *Circ: Cardiovascular Imaging* 2019 [44]	Radiomics analysis was able to differentiate between pannus and other abnormalities in periprosthetic masses
	Qian et al. *BMC Cardiovasc Disord* 2021 [45]	Radiomics features could differentiate between cardiac thrombi and cardiac myxomas
	Chun et al. *Eur Radiol* 2021 [46]	Radiomics features differentiated between thrombus and statis in filling defects in the left atrial appendage
**Radiomics analysis of periaortic adipose tissue**	Tharmaseelan et al. *Int J Cardiovasc Imaging* 2022 [10]	7 radiomics features of periaortic adipose tissue revealed a prediction of the presence of local aortic calcification

## Data Availability

Not applicable.
